# Influence of Perturbation Velocity on Balance Control in Parkinson’s Disease

**DOI:** 10.1371/journal.pone.0086650

**Published:** 2014-01-22

**Authors:** Lars B. Oude Nijhuis, John H. J. Allum, Wandana Nanhoe-Mahabier, Bastiaan R. Bloem

**Affiliations:** 1 Radboud University Nijmegen Medical Centre, Donders Institute for Brain, Cognition and Behaviour, Department of Neurology, Nijmegen, The Netherlands; 2 Division of Audiology and Neurootology, Department of ORL, University Hospital, Basel, Switzerland; Florey Institute of Neuroscience & Mental Health, Australia

## Abstract

Underlying somatosensory processing deficits of joint rotation velocities may cause patients with Parkinson’s disease (PD) to be more unstable for fast rather than slow balance perturbations. Such deficits could lead to reduced proprioceptive amplitude feedback triggered by perturbations, and thereby to smaller or delayed stabilizing postural responses. For this reason, we investigated whether support surface perturbation velocity affects balance reactions in PD patients. We examined postural responses of seven PD patients (OFF medication) and eight age-matched controls following backward rotations of a support-surface platform. Rotations occurred at three different speeds: fast (60 deg/s), medium (30 deg/s) or slow (3.8 deg/s), presented in random order. Each subject completed the protocol under eyes open and closed conditions. Full body kinematics, ankle torques and the number of near-falls were recorded. Patients were significantly more unstable than controls following fast perturbations (26% larger displacements of the body’s centre of mass; P<0.01), but not following slow perturbations. Also, more near-falls occurred in patients for fast rotations. Balance correcting ankle torques were weaker for patients than controls on the most affected side, but were stronger than controls for the least affected side. These differences were present both with eyes open and eyes closed (P<0.01). Fast support surface rotations caused greater instability and discriminated Parkinson patients better from controls than slow rotations. Although ankle torques on the most affected side were weaker, patients partially compensated for this by generating larger than normal stabilizing torques about the ankle joint on the least affected side. Without this compensation, instability may have been greater.

## Introduction

Parkinson’s disease (PD), in the early stages, is characterized by unilaterally occurring symptoms, such as resting tremor and bradykinesia. As the disease progresses, axial symptoms, such as gait disability and postural instability, become apparent [1], [2]. Postural instability and the resulting falls are considered cardinal features that occur in over 70% of PD patients per year, and which commonly result in injury, fear of falling, social isolation and immobilization [3], [4]. A better understanding of the underlying pathophysiological processes could serve as a basis for improved treatment strategies [5]. However, assessing postural instability in PD remains a challenge, due to the complexity and variety of the balance control mechanisms involved [4]. Moreover, currently available clinical measures are not sensitive enough to detect early symptoms of postural instability in PD patients, which can only be detected until a relatively late stage in the progression of the disease [3]. Therefore, alternative measures should be sought to identify fallers in earlier stages of PD.

Dynamic posturography, a technique that uses experimentally induced balance perturbations via support surface rotations or translations, offers several advantages over clinical measures [5]. For example, the specific parameters of the balance disturbance can be controlled and standardized. Furthermore, specific elements of postural control can be selectively manipulated to render the balancing task more challenging. The technique also allows for detailed and objective analysis. At a group level, posturography techniques provide reliable diagnostic indicators, provided test protocols are fitted to clinically-rated impairments [5], [6]. For example, vestibular loss subjects are only unstable in response to slow movements of the support surface (ca. 5 deg/s) when standing with eyes closed, but with both eyes open, they are also unstable in response to fast (60 deg/s) support-surface rotations [7]. The slow perturbations normally produce a sensation of movement, but this is below the threshold of detection in vestibular loss subjects due to imbalanced background activity in the vestibular nuclei [8]. When visual inputs are present, falling is avoided following an upgrading of visual sensory information [9]. For fast platform movements visual inputs are insufficiently rapid and the loss of vestibular sensory gain causes vestibular loss subjects to respond as if the stimulus was smaller and fall [7]. Thus, both slow and fast rotations can cause instability in vestibular loss subjects, but fast rotations provide a better diagnostic yield [10].

In PD patients, fast perturbations may also bring about more instability compared to slow perturbations, particularly in light of increasing evidence for somatosensory processing defects in this disorder [11], [12], because proprioceptive inputs are essential for triggering automatic postural responses to fast perturbations [7], [13]. However, slow perturbations may also cause instability because 40% of PD patients experience spontaneous movement sensations despite having a normal neurological examination [14]. Thus, it is an open question which velocity of support surface movements during stance leads to a better diagnosis of impaired balance control in PD patients.

We investigated this question, by testing whether support surface perturbation velocity affects postural control in PD patients with respect to healthy controls. We hypothesized that differences in postural control between patients and controls, as expressed by displacement of the body’s centre of mass following support surface movements, would be greater during fast perturbations, due to processing difficulties with proprioceptive inputs that are normally needed to generate correcting forces to keep the body upright. Moreover, if there was no visual feedback, PD patients would have to rely more on deficient proprioceptive sensory feedback. Therefore, we also hypothesized that any observed differences would be more pronounced when visual feedback was lacking.

## Materials and Methods

### Ethics Statement

A neurologist specialized in movement disorders established that all subjects had the capacity to consent. Prior to participation in the experiments, all subjects gave written informed consent according to the Declaration of Helsinki. The local ethical committees of the Radboud University Nijmegen Medical Centre and the University Hospital Basel approved the study.

### Participants

We examined seven patients diagnosed with PD (according to the UK Brain Bank criteria [15]) and eight matched healthy controls (table 1). Patients were selected based on having moderate disease severity (Hoehn and Yahr stage range 2–3, as measured during a practically defined OFF state, more than 12 hours after intake of dopaminergic medication). Despite the moderate disease severity, patients were able to stand independently throughout the course of the experiment. Patients were tested in a practically defined OFF state as dopaminergic medication may partially influence elements of postural control [16]. Patients were characterized clinically using the Unified Parkinson’s Disease Rating Scale (UPDRS) [17]. In addition, functional balance was assessed using the Tinetti Mobility index [18] and fall history was noted. Balance confidence was determined using a short version of the Activities-specific Balance Confidence (ABC) scale [19], the ABC-6 (see table 1) [20]. Routine neurological examination showed no clinical proprioceptive deficits in patients. Furthermore, the PD patients had no significant postural tremor, marked cognitive impairment, or inability to comply with the test instructions. Subjects had no other causes of balance impairment.

**Table 1:** Subject characteristics.

	Patients	Control subjects
Demography		
Number of subjects	7	8
Men/women	6/1	7/1
Age (y)	56.1±8.6	53.4±7.0
Height (cm)	179±7	178±0.09
Weight (kg)	79.1±16.7	79.8±14.5
Balance and gait scores		
Tinetti Balance	2.3±2.2	0.0±0.0
Tinetti Gait	4.3±2.1	0.0±0.0
Balance confidence(ABC-6) (%)	68.8±16.8	93.3±1.7
Fall history		
Fallers	1 (14.3%)	n.a.
Falls in the past year	12	n.a.
PD related variables		
Disease duration (y)	5.0±1.8	
Hoehn and Yahr	2.6±0.2	
UPDRS-total (off medication)	56.6±21.6	
UPDRS-III (motor, off medication)	41.0±17.1	

Data is shown as mean ± standard deviation. Clinical characterization using the Tinetti mobility index [18]; ABC-6 = Short Activities-specific Balance Confidence [20]; H&Y = Hoehn & Yahr, range 0 (no signs of disease) to 5 (wheelchair bound/bedridden) [37]; UPDRS = Unified Parkinson’s Disease Rating Scale [17].

### Experimental Protocol

Balance control was assessed using previously described techniques [21]–[23]. Participants stood barefoot on a servo-controlled dual-axis platform with their arms hanging by their sides. The feet were strapped to the platform with the ankles aligned with the pitch axis of rotation. Thus subjects used an in-place balance correcting reaction and no stepping movements to correct for the support-surface tilt. Stance width was the same for all subjects (14 cm).

#### Platform rotations

Subjects were tilted 24 times by the support-surface platform at a constant amplitude of 7.5 deg, all in the backward direction. Platform tilts occurred at three different velocities 60 deg/s (FAST); 30 deg/s (MEDIUM); and 3.8 deg/s (SLOW) that were delivered in random order. Thus, subjects received eight backward perturbations at each velocity. The protocol was completed under both eyes open (EO) and eyes closed (EC) conditions with presentation order counterbalanced across subjects. Prior to starting the experiment, subjects received at least 5 practice trials that were excluded from further analysis to reduce first trial and habituation effects affecting the data [23], [24]. Each subsequent perturbation was preceded by a random 5–11 sec delay, during which visual feedback of the participants’ own anterior–posterior and medial–lateral ankle torques was presented to the participant on a cross with rows of light- emitting diodes positioned 4 m in front of the subject. The visual feedback was used to standardize the prestimulus position of participants across trials and a stimulus was not presented until ankle torque was within a range of 4 Nm of that of the subject’s initial standing position. To set this level we asked subjects to stand comfortably. However, equal loading was not controlled.

### Outcome Measures

We recorded kinematic and kinetic responses. To collect full body kinematics, we instrumented participants with 18 infrared emitting diodes (IREDs) [23]. The IREDs were placed bilaterally on the following anatomical landmarks: frontally at the level of the malleoli, at the centre of the patellae, frontally at the level of the greater trochanters, anterior superior iliac spine, elbow axis, acromion, processus styloïdeus, temple, one at the chin, and one at the sternal angulus. Three additional IREDs, placed at the front corners and centre of the platform surface, were used to track platform movements. The Optotrak motion analysis system (Northern Digital Canada Inc., Waterloo) tracked the IREDs with a frequency of 64 Hz. Ankle torques were calculated on-line from the vertical forces measured with strain gauges mounted under each corner of a foot plate supporting each foot. That is, forces under each foot were measured independently. The anterior-posterior (AP) torques of each foot were calculated with respect to the pitch axis of the rotating platform and medial-lateral torques with respect to the mid-line of each foot plate. The kinetic responses, used AP ankle torques, were sampled at 1024 Hz. All recordings were initiated 100 ms prior to the onset of platform rotation and had a sampling duration of 3 s.

### Data Analysis

#### Kinematic analyses

**Figure 1:**
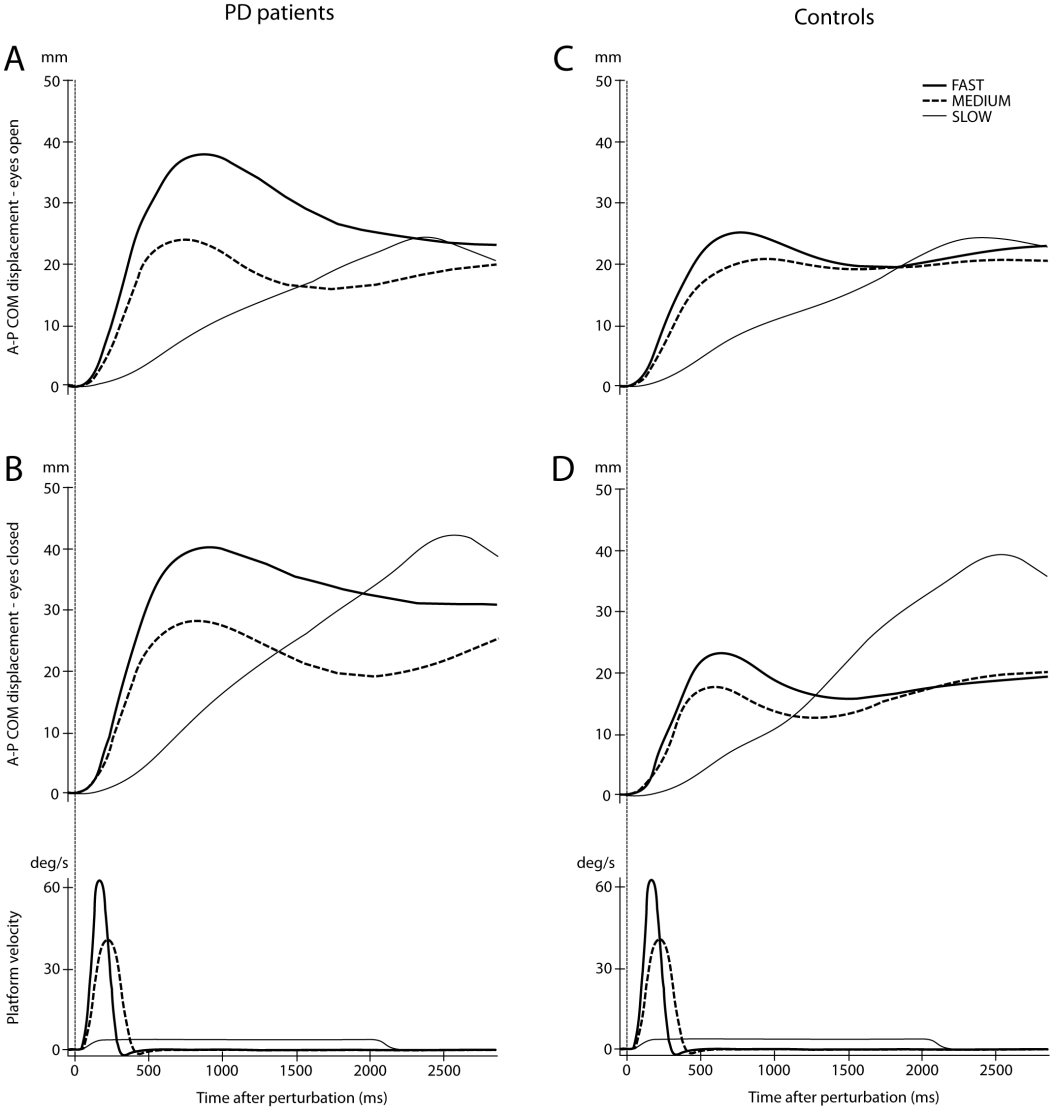
Population average centre of mass (COM) displacements. (A) Traces of the anterior-posterior displacement of the COM (mm) to FAST, MEDIUM, and SLOW rotations in PD patients with eyes open. (B) Traces as in A, for PD with eyes closed. (C) Traces as in A for controls with eyes open. (D) Traces as in C for controls with eyes closed. In the lower panels traces of the platform velocities (deg/s) for FAST, MEDIUM and SLOW rotations are shown. The vertical lines at 0 ms represent the onset of the platform rotation.

For each trial we used the IREDs movements to calculate anterior-posterior (AP) displacement of the body COM (see figures 1A–1D), using a previously described model of the body [22], [25]. We calculated the amplitude of backward COM displacement as the area under the curve (AUC) of COM movement, as our main outcome measure, using trapezoid integration from stimulus onset (0 ms) to the end of the recording (2800 ms) [23].

#### Kinetics

**Figure 2:**
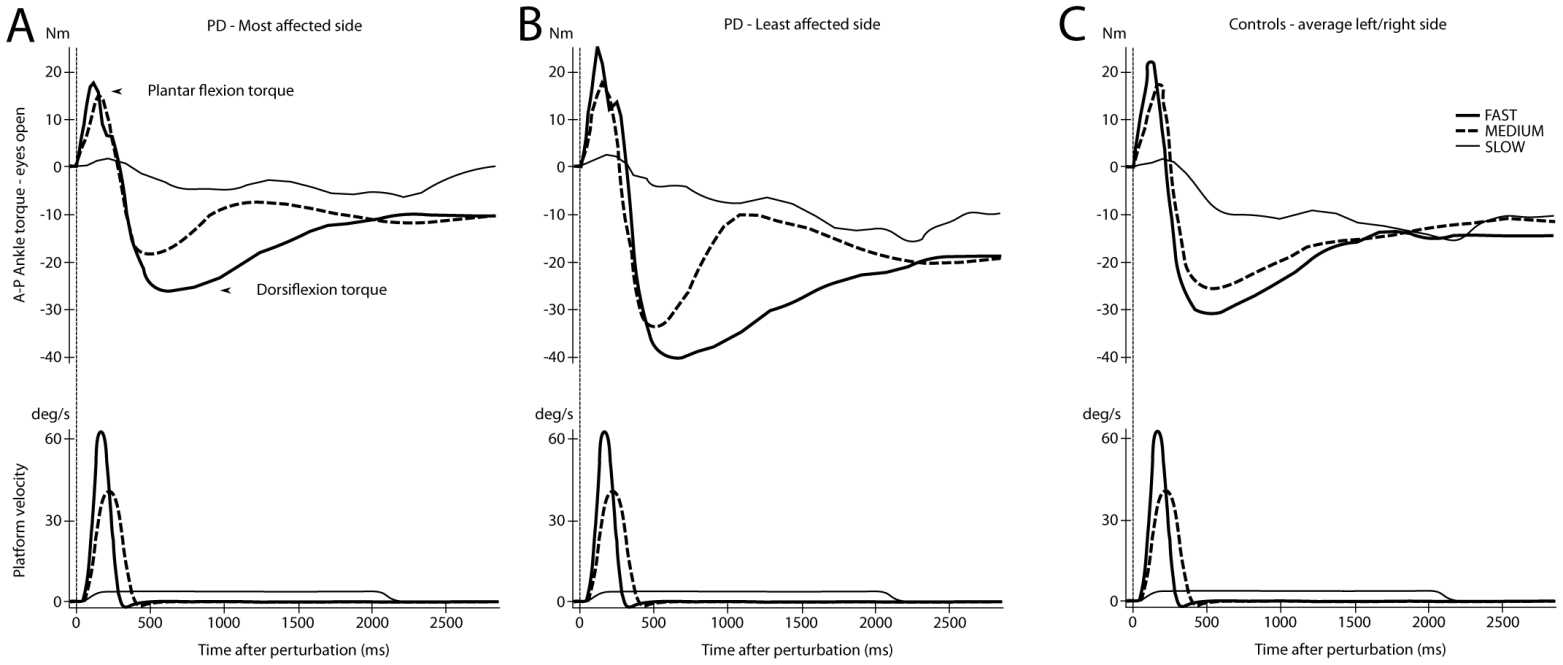
Single subject ankle torque. (A) Anterior-posterior ankle torque traces to FAST, MEDIUM and SLOW perturbations on the most affected side of a PD patient with eyes open. (B) Traces as in A, for the least affected side of a PD patient with eyes open. (C) Average traces of anterior-posterior ankle torque on the left and right side of a control with eyes open. In the lower panels traces of the platform velocities (deg/s) for FAST, MEDIUM and SLOW rotations are shown. The vertical lines at 0 ms represent the onset of the platform rotation.

The AP ankle torque signals were biphasic (see figure 2A–2C). Hence we measured the maximum ankle torque amplitude (of plantar flexion or positive ankle torque) and the minimum value (of dorsiflexion or negative torque). An averaging interval of 50 ms around the maximum and minimum values was used as the analysis measure for population comparisons. The torque outcomes were calculated separately for the left and the right ankle. For PD patients, left and right ankle torques were defined as the least and most affected side according to their clinical examination.

#### Near-falls

The number of the near-falls was registered as well as the trial stimulus velocity which elicited the fall. A response was defined as a near-fall when the subject required to grasp handrails or be helped by a spotter to prevent a fall. All near-falls occurred after the trial recording duration of three seconds.

### Statistical Analyses

Our primary analyses concentrated on between-groups comparisons of PD patients and controls using a repeated measures analyses of variance (ANOVA) model for group (PD patients/controls) and two within-subject factors; vision (eyes open/eyes closed) and velocity (FAST/MEDIUM/SLOW) for both kinematic and kinetic data-values. For kinetic data-values, we first used two repeated measures analyses for the within-subject factors vision, velocity and side, to determine differences in ankle torques between both the most and least affected side in PD patients and the left and right sides in controls. Values were back-transformed into percentages using the mean of the control values for FAST stimuli, specific to stimulus order as the normalizing factor. Values were then expressed as mean ±95% confidence intervals (95% CI). Before these analyses, we ascertained that the data values were normally distributed. Significant main and interaction effects were further explored using post-hoc tests. These consisted of Student’s paired t-tests, for which significance levels set at 0.05 were adjusted downward (Bonferroni correction), according to the number of comparisons.

Furthermore, we used a univariate analysis (General linear model) with the COM displacement for FAST as the dependent variable, group (PD or controls) as fixed factor and the COM displacement for velocity as covariate, to test if the difference between PD and controls was larger for FAST compared to SLOW perturbations. The relationships between balance control scores (center of mass and ankle torques) and disease severity (measured with the total UPDRS score) were characterized by Pearson’s correlation coefficients.

## Results

### Effect of Perturbation Velocity on COM Displacement

Table 2 presents the estimated marginal means ±95% confidence intervals. The amplitude of COM displacement (as defined by the area under the COM curve) was significantly larger for PD patients, compared with controls (main effect of group F_(1, 89)_ = 10.54, P<0.01). Furthermore, this difference was significantly affected by the rotation velocity of the support-surface platform. The repeated measures analysis revealed both a main effect of velocity (F_(1.83, 163.07)_ = 54.88, P<0.01), such that COM displacement was larger for fast perturbations, and a group × velocity interaction effect (F_(1.83,163.07)_ = 19.46, P<0.01), such that PD patients had more differences between both velocity conditions than controls did.

**Table 2:** Area under curve of COM displacement.

		*Patients*	*Controls*	*Patients*	*Controls*
	*Velocity*	*eyes open*	*eyes open*	*eyes closed*	*eyes closed*
Area under curve of COM displacement	*FAST*	8.47 (7.71–9.22)	5.96 (4.88–7.04)	9.90 (8.27–11.52)	7.03 (6.07–8.00)
	*MEDIUM*	6.20 (5.38–7.02)	5.03 (4.08–5.98)	6.43 (5.22–7.63)	6.35 (5.61–7.08)
	*SLOW*	4.99 (4.13–5.84)	4.04 (3.26–4.82)	6.78 (5.56–8.01)	6.55 (5.41–7.69)

Estimated marginal means (95% confidence intervals) for the total area under the curve of the COM displacement (mm.s×10^4^). The table summarizes results of the repeated measures ANOVA as function of group, vision and velocity, comparing PD patients and controls for FAST, MEDIUM and SLOW. Estimated marginal means were taken from the ANOVA analysis (see comments on general linear model in the Methods section).

Without vision, COM displacement across both groups was 17% larger, as compared with eyes open (F_(1.00, 89.00)_ = 21.72, P<0.01). However, vision did not interact significantly with either group (F_(1, 89)_ = 0.44, P = 0.51) or velocity, although the visual effect tended to be greater for fast perturbations (F_(1.81, 160.88)_ = 2.82, P = 0.068). Therefore, we pooled the results of the eyes open and eyes closed conditions for further analysis of COM displacement.

Both patients (F_(1.57,48.80)_ = 38.12, P<0.01) and controls (F_(2, 116)_ = 9.82, P<0.01; figure 1A–1D) were more unstable during FAST than SLOW rotations, as reflected by the significantly larger COM displacements during FAST compared to SLOW rotations. For PD patients COM displacement was 38% (95%CI = 27–49%) larger for FAST compared to SLOW rotations (P<0.01). For controls COM displacement was 16% (95%CI = 9–24%) larger during FAST compared to SLOW (P<0.01).

Patients were significantly more unstable than controls during FAST perturbations (26% larger COM displacement, 95%CI = 16–37%, P<0.01), but not during MEDIUM and SLOW perturbations. Thus for SLOW rotations this group difference was 9% (95% CI = 6–24%).

### Effect of Perturbation Velocity on Anterior-posterior Ankle Torque

For PD patients, ankle torques are reported separately for the most affected side and the least affected side, because we expected to record asymmetrical responses. Indeed, a within-group analysis (velocity×side×vision) showed significant main effects of side for both the maximum plantar flexion torque (F_(1, 55)_ = 6.84, P<0.05) and the minimum dorsiflexion torque (F_(1, 55)_ = 111.37, P<0.01). The latter represents the amplitude of stabilizing torque.

In controls, a within-group analysis (velocity×side×vision) showed no significant effects of side for both the peak plantar flexion torque and dorsiflexion torque. Therefore, the results for the left and right anterior-posterior ankle torques were averaged together.

#### Ankle plantar flexion torque

The early maximum of plantar flexion torque was not significantly influenced by group (F_(2,173)_ = 1.09, P = 0.34). We did not record a significant interaction effect for group × vision × velocity (F_(3.73,316.04)_ = 0.83, P = 0.50) either. A main effect of velocity was found (F_(1.91, 330.25)_ = 1296.32 P<0.01) with significantly larger plantar flexion torques for higher platform rotation velocities (table 3, figure 2A–2C).

**Table 3:** Ankle torque amplitude.

			*Patients*		*Controls*
	*Vision*	*Velocity*	Most affected side	Least affected side	Average left and right
Plantar flexion torque	*Eyes open*	*FAST*	18.8 (17.6–19.9)	19.7 (18.0–21.5)	16.8 (15.6–18.1)
		*MEDIUM*	15.0 (13.9–16.0)	15.3 (13.9–16.7)	13.7 (12.7–14.7)
		*SLOW*	2.5 (2.2–2.9)	2.7 (2.2–3.2)	2.2 (1.8–2.6)
Dorsiflexion torque		*FAST*	−27.5 (−29.8–25.1)	−39.6 (−42.4–36.9)	−28.5 (−29.9–27.1)
		*MEDIUM*	−20.7 (−22.3–19.0)	−34.8 (−37.7–31.8)	−26.2 (−27.5–24.8)
		*SLOW*	−16.2 (−18.5–13.9)	−36.1 (−40.2–32.1)	−23.1 (−22.0–28.7)
Plantar flexion torque	*Eyes closed*	*FAST*	18.9 (17.9–19.9)	20.1 (18.6–21.6)	19.6 (17.9–21.3)
		*MEDIUM*	14.5 (13.4–15.5)	15.3 (13.8–16.8)	16.4 (14.9–17.9)
		*SLOW*	2.4 (2.0–2.9)	3.2 (2.6–3.9)	37.8 (31.3–44.3)
Dorsiflexion torque		*FAST*	−28.8 (−31.2–26.5)	−40.1 (−42.6–37.5)	−32.0 (−33.8–30.2)
		*MEDIUM*	−23.1 (−25.0–21.3)	−35.5 (−38.3–32.7)	−31.0 (−32.5–29.4)
		*SLOW*	−24.3 (−26.5–22.1)	−37.5 (−41.0–34.0)	−29.6 (−31.6–27.6)

Estimated marginal means (95% confidence intervals) of the plantar and dorsiflexion torque amplitudes Nm. The table summarizes results of the repeated measures ANOVA as function of group, vision and velocity, comparing PD patients and controls for FAST, MEDIUM and SLOW.

#### Ankle dorsiflexion torque

The minimum value of (stabilizing) dorsiflexion torque was significantly different between groups (PD-most affected side compared to PD-least affected side compared to controls) (F_(2, 173)_ = 55.36, P<0.01) and this difference was influenced by both vision and velocity (F_(3.70, 319.73)_ = 3.86, P<0.01).

In all three groups, ankle dorsiflexion torque amplitudes were significantly larger for FAST compared with MEDIUM and SLOW rotations, during the eyes open condition (table 3, P<0.01). Furthermore, the difference in ankle dorsiflexion torque magnitudes between the least affected side of PD patients and controls was larger for FAST compared with SLOW rotations both during eyes open (69%, 95%CI = −49–188) (univariate analysis, F_(1,54)_ = 50.12, P<0.01) and eyes closed (101%, 95%CI = 16–186) (univariate analysis, F_(1, 54)_ = 36.10, P<0.01).

In contrast, the difference in ankle dorsiflexion torque between the most affected side of PD patients and controls was significantly larger for SLOW compared to FAST rotations, but only during eyes closed trials (63%, 95%CI = −55–183) (F_(1, 54)_ = 17.78, P<0.01).

During FAST rotations ankle dorsiflexion torque showed a main effect for group (PD-most affected side vs. PD-least affected side vs. controls) during both eyes open (F_(2, 110)_ = 40,22, P<0.01) and eyes closed (F_(2,110)_ = 31.08, P<0.01). Ankle dorsiflexion torque was significantly larger on the least affected side of PD patients compared with controls (P<0.01). However, dorsiflexion torque on the most affected side, was smaller compared with controls (P<0.01) (see table 3).

### Near-falls

We recorded more near-falls during FAST compared to SLOW rotations. In two PD patients we recorded a total of five near-falls during FAST rotations. In two other patients we recorded one near-fall each during SLOW rotations. One control subject showed two near-falls during FAST rotations. All near-falls were recorded during the eyes closed condition.

### Relationship between Disease Characteristics and Balance Control Scores

FAST rotations were most discriminative between PD patients and controls. Therefore, we correlated balance control scores (COM and ankle torques) during FAST rotations to clinical scores of PD patients. COM displacement during FAST rotations did not correlate with UPDRS total scores. However, the amplitude of dorsiflexion torque showed high positive correlations with total UPDRS scores (suggesting worse stabilization for the more affected patients) on the least affected side of PD patients and in the eyes open condition (r = 0.86, P = 0.01). Significant correlations were not seen for the most affected side.

## Discussion

We studied differences in balance control between PD patients and controls following perturbations to upright stance driven by support surface rotations of different velocities. Fast rotations with eyes open were most discriminative for postural instability (measured as COM displacement amplitude) between PD patients and controls. Fast platform rotations led to more instability in both PD patients and controls, compared to slow perturbations. Absence of visual feedback significantly increased instability for all rotation velocities in PD patients, but only during slow rotations in controls.

During fast rotations, stabilizing dorsiflexion torques were significantly larger on the least affected side of PD patients compared with the most affected side and with controls. This difference between sides in PD patients suggests the presence of a compensating mechanism. Furthermore, ankle dorsiflexion torque on the least affected side was highly correlated with disease severity (smaller ankle torques with a more severe disease state), suggesting that this compensation declines with disease progression.

### Stimulus Intensity Effects on Balance Control

One obvious explanation for the larger instability during fast perturbations compared to slow perturbations is the higher stimulus intensity during fast rotations. We used a platform rotation of 7.5 deg for all conditions. Therefore, the acceleration of our slow perturbations was smaller compared to the fast perturbations. PD symptoms such as bradykinesia may cause patients to have more difficulty to respond adequately to the fast perturbations.

### Compensatory Forces in the Least Affected Leg during Fast Perturbations

Ankle torques associated with stabilization of upright posture, were smallest on the most affected side of PD patients following fast perturbations. This points to a reduced force production in the most affected leg of PD patients [26], [27]. However, we recorded large dorsiflexion torques on the least affected side, and these torques were even larger compared with the dorsiflexion torques generated by controls. This suggests that the torques generated on the least affected side may compensate for the decreased force production on the most affected side, similar to balance recovery in both stroke patients who use their non-paretic leg to stabilize posture following external perturbations [28], [29] and with balance control after lower limb amputation [30]. However, in our study ankle dorsiflexion torques on the least effected side showed high correlations with disease severity during fast perturbations. Thus, more severely affected patients showed decreased ankle dorsiflexion torques on the least affected side, presumably because their ability to compensate for the impaired force generation on the most affected side may decline when the disease manifests itself more and more bilaterally. Therefore, it remains to be seen whether ankle torque asymmetries can be used as an outcome measure for interventions aimed at improving balance in PD.

### Instability Following Slow Perturbations

We hypothesized that fast support-surface rotations would lead to increased instability and better discrimination between patients and controls than slow ones. However, there are reasons to suggest otherwise. The slow perturbations used in this experiment had a longer period of constant velocity displacement compared to the fast perturbations. Previous reports showed that the rapid deceleration terminating fast translational perturbations can be used by both healthy young and elderly subjects to help them in staying upright [31], [32]. A delay of the platform deceleration phase, as occurs with SLOW rotational perturbations, leads to greater late COM instability with eyes closed in both PD patients and control subjects (see traces at 2.5 s in figure 2A–2C). However, the net difference in COM displacement between populations was greatest for fast rotations.

During fast perturbations, balance reactions rely more on early sensory proprioceptive feedback to adequately trigger balance reactions to the platform movements [7], [13]. This early feedback leads to responses that may be split into an early short-latency response more dependent on stimulus acceleration and a later medium latency response more dependent on stimulus velocity [33]. The latter is increased in PD [34], suggesting a false overestimation of velocity leading to an increased activation of triceps muscle and reduced stabilizing ankle torque. While this would be a parsimonious explanation of our findings it should be recalled that medium and long latency responses are under subcortical, presumably reticulospinal, control. For example, the reticulospinal reflex pathway is affected in Parkinson’s disease leading to modification of spinal interneurone activity [35]. Acitivity of Ia inhibitory interneurones is facilitated and activity of Ib inhibitory interneurones is decreased [36], which could also enhance medium latency reflexes.

Recent reports have shown that PD patients have impaired axial kinesthesia; this is commonly defined as the ability to detect joint motion or a change in the position of a joint at low velocities (1°/s) [11]. Such axial kinesthesia could have led to increased instability in PD patients during slow perturbations. In comparison, the velocity of 3.8 deg/s used for SLOW perturbations in our study may not have been slow enough to reveal an influence of such impaired axial kinesthesia. For these velocities of 3.8°/s, the question also arises if threshold deficits are then due to proprioceptive or vestibular sensory deficits [8].

## Conclusions

Fast balance perturbations caused greater instability and discriminated Parkinson patients better from controls than slow perturbations. Stabilizing torques generated about the least affected ankle compensated for decreased torques about the most affected ankle, suggesting that aiding this compensation process may be useful for prevention of falls in PD. However, this compensation process deteriorated with increased disease severity.
